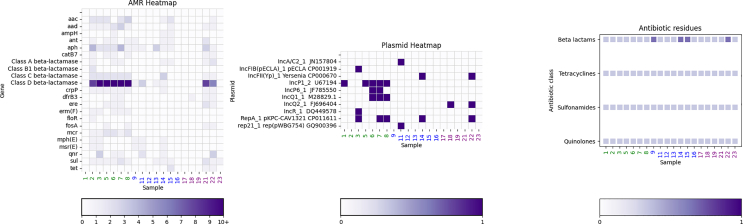# The drainome: longitudinal metagenomic characterization of wastewater from hospital ward sinks to characterize the microbiome and resistome and to assess the effects of decontamination interventions

**DOI:** 10.1016/j.jhin.2024.06.005

**Published:** 2024-11

**Authors:** L.B. Snell, D. Prossomariti, A. Alcolea-Medina, M. Sasson, M. Dibbens, N. Al-Yaakoubi, G. Humayun, T. Charalampous, C. Alder, D. Ward, A. Maldonado-Barrueco, O. Abadioru, R. Batra, G. Nebbia, J.A. Otter, J.D. Edgeworth, S.D. Goldenberg

**Affiliations:** a Department of Infectious Diseases, King's College, London, UK; b Directorate of Infection, Guy's and St Thomas' NHS Foundation Trust, London, UK; c Infection Sciences, Synnovis Analytics LLP, London, UK

**Keywords:** Drainome, Drain microbiome, Wastewater surveillance

## Abstract

**Background:**

Hospital drains and water interfaces are implicated in nosocomial transmission of pathogens. Metagenomics can assess the microbial composition and presence of antimicrobial resistance genes in drains (‘the drainome’) but studies applying these methods longitudinally and to assess infection control interventions are lacking.

**Aim:**

To apply long-read metagenomics coupled with microbiological measurements to investigate the drainome and assess the effects of a peracetic-acid-containing decontamination product.

**Methods:**

Twelve-week study in three phases: a baseline phase, an intervention phase of enhanced decontamination with peracetic acid, and a post-intervention phase. Five hospital sink drains on an intensive care unit were sampled twice weekly. Each sample had: (1) measurement of total viable count (TVC); (2) metagenomic analyses including (i) taxonomic classification of bacteria and fungi (ii), antibiotic resistance gene detection, (iii) plasmid identification; and (3) immunochromatographic detection of antimicrobial residues.

**Findings:**

Overall TVCs remain unchanged in the intervention phase (+386 cfu/mL, SE 705, *P* = 0.59). There was a small but significant increase in the microbial diversity in the intervention phase (–0.07 in Simpson's index, SE 0.03, *P* = 0.007), which was not sustained post-intervention (–0.05, SE 0.03, *P* = 0.08). The intervention was associated with increased relative abundance of the *Pseudomonas* genus (18.3% to 40.5% (+22.2%), SE 5.7%, *P* < 0.001). Extended spectrum β-lactamases were found in all samples, with NDM-carbapenemase found in three drains in six samples. Antimicrobial residues were detected in a large proportion of samples (31/115, 27%), suggesting use of sinks for non-handwashing activities.

**Conclusion:**

Metagenomics and other measurements can determine the composition of the drainome and assess the effectiveness of decontamination interventions.

## Introduction

Nosocomial infections involving transmission of pathogens are common, are associated with excess morbidity and mortality, and with significant healthcare costs [[1], [2], [3]]. Transmission of these organisms can occur person-to-person; however, there is increasing recognition that environment-to-person transmission plays a significant role in nosocomial infection [4]. Nosocomial infections are also associated with resistant organisms [5].

Hospital sinks and associated drainage systems are one such environmental nidus of organisms capable of infecting humans and harbouring antimicrobial resistance genes (ARGs), with recent studies linking nosocomial infection to hospital sinks [[6], [7], [8]]. Increased efforts have therefore focused on characterizing the microbiome of sink outflow and reducing it using various decontamination strategies [9]. Some ICUs in the Netherlands have implemented ‘water-free’ ICUs to avoid nosocomial transmission from sinks [10].

Previous studies have used traditional microbiological methods to characterize the microbial community in sink wastewater, or to identify antimicrobial residues present in the drains [11,12]. Only small studies using metagenomics to investigate wastewater microbiome have been published, typically as a proof of concept, and without longitudinal genomic data, or unlinked to interventions [9,[12], [13], [14]].

Our aim was therefore to perform a longitudinal study to combine microbiological measurements with metagenomic analysis to characterize the microbiome and resistome from sink wastewater in our hospital. Colloquially we refer to the composition of the microbiome and resistome of this sink wastewater as the ‘drainome’. During this longitudinal period we instigated a period of enhanced decontamination using a peracetic-acid-based product to assess its effect on the drainome.

## Methods

### Study protocol

The study was performed on a 15-bed intensive care unit at St Thomas' Hospital (part of Guy's and St Thomas' NHS Foundation Trust, London, UK). The study ran over 12 weeks between July and October 2023, separated into three equal phases lasting four weeks each: (i) baseline phase; (ii) intervention phase of enhanced decontamination; (iii) post-intervention phase. Five clinical handwash basins were included. The sink drains are named after their location on the ward: ‘bed 5’, ‘bed 9’, ‘bed 11’, ‘bed 14’ and ‘side room (sr) 4’. A representative photograph showing the sink design is provided in the Supplementary Appendix (Methods). Samples were collected twice a week. On one date in the post-intervention phase, samples could not be collected (*n* = 5).

During the intervention phase, sinks were subjected to enhanced decontamination with Clinell® Drain Disinfectant (Gama Healthcare, Hemel Hempstead, UK), which contains peracetic acid as the active ingredient. The disinfectant product was applied during the intervention phase according to manufacturers' instructions, initially on three consecutive days and then twice per week thereafter. The disinfectant was allowed to dwell in the p-trap for a minimum of 15 min before the sink was used; this was facilitated by direct observation and the use of indicator tape over the sink instructing staff that the sink was temporarily out of use.

A minimum of 20 mL of wastewater was then aspirated from the p-trap of each drain. A flexible Ryles tube was slowly guided into the drain whilst simultaneously drawing back on a sterile syringe until water was aspirated. The Ryles tube was repositioned as necessary. The wastewater was stored in sterile Falcon tubes.

Each drain water sample was subjected to: (1) measurement of total viable count (TVC); (2) metagenomic analyses including (i) taxonomic classification of bacteria and fungi, (ii) antibiotic resistance gene detection, (iii) plasmid identification; and (3) immunochromatographic detection of antimicrobial residues.

### Measurement of total viable count and detection of antibiotic residues

TVC was estimated using the MicroSnap™ TVC assay (Hygiena, Watford, UK). This non-culture method utilizes 1 mL of wastewater incubated in a proprietary device used according to manufacturer's instructions. After 7 h of incubation at 30 °C, TVC is estimated in cfu/mL using the Hygiena luminometer and measured in relative light units. During the implementation phase, TVC samples were taken immediately before application of the disinfectant product.

Antimicrobial residues were measured using the immunochromatographic QuaTest BTSQ 4-in-1 kit (Ringbio, Beijing, China) as previously validated, giving a qualitative result of present (positive) or absent (negative) for four antimicrobial classes: β-lactams (B), tetracyclines (T), sulfonamides (S), and quinolones (Q) [12].

### Metagenomic processing and bioinformatic analysis

Processing for metagenomics used an adapted version of our previously published method [15]. Briefly, 20 mL of sink wastewater was centrifuged for 10 min at 4500 *g*. The pellet was resuspended in 330 μL bacterial lysis buffer (Qiagen, Manchester, UK). This mixture was then bead-beat for 3 min at 50 oscillations/s in Lysis Matrix E beads (MP Biomedicals, Irvine, CA, USA). After centrifugation at max speed for 1 min, nucleic acid was extracted from the pellet (MagNA Pure 24 System; Roche, Welwyn Garden City, UK). The extract was prepared for sequencing using SQK-RPB004 as previously described on the GridION (Oxford Nanopore Technologies, Oxford, UK) using R9.4.1 flow cells [15]. No template, water controls were run to provide information on background laboratory contaminants. Negative control subtraction, where taxonomic reads found in the negative control are removed from samples in the same run, was employed to account for contaminants introduced into samples during sample processing [16].

Basecalling and demultiplexing of reads was performed using MinKNOW (v5.2.4) with Guppy (v6.2.4) using high-accuracy basecalling model and utilizing the default-length filter of 200 base pairs and barcodes-at-both-ends to prevent barcode crosstalk. Reads were then analysed with epi2me-labs wf-metagenomics (v2.8.0-g4397263; Oxford Nanopore Technologies), which classifies reads using Bracken against the PlusPF-8 database for taxonomic classification, against the ResFinder database for detection of antimicrobial resistance genes, and against the PlasmidFinder database for mobile genetic elements [[17], [18], [19]]. After taxonomic classification, relative abundance at the level of genus or species was calculated. For detection of antimicrobial resistance genes, including plasmids, a threshold nucleotide identity of 90% and gene coverage of 90% was used, based on our previous validation in clinical samples [15]. The presence of ARGs and plasmids was reported in a binary fashion: detected or not detected, as inter-sample and inter-run factors preclude direct comparison of counts of specific genes or elements.

The output from wf-metagenomics was further processed using bespoke bash and python scripts. Plots were created in python using pandas v2.2.1, numpy v1.26.4, matplotlib v3.8.3, and seaborn v0.13.2.

### Statistical analysis

To investigate the impact of different treatment phases on outcome measures, linear mixed effects models fitted through maximum likelihood estimation were employed. This approach accounted for repeated measurements taken from the same drains across the three distinct study phases. This model treated the different phases of treatment as fixed effects to assess their impact on outcomes. A random effect parameter for each drain was included to account for the inherent intra-drain variability that could influence outcomes. The same approach was used for testing changes in the TVC, Simpson's diversity and proportion of genera (e.g. *Pseudomonas* sp.) in samples across study phases. Model coefficients are presented with standard errors (SE) and with a significance threshold set at *P* < 0.05. Statistical testing was performed in python using statsmodels v0.14.1.

## Results

Across the study period, 115 samples were collected from five sink drains, with 40 collected in the baseline phase, 40 in the intervention phase, and 35 in the post-intervention phase. Of 115 samples, 108 (94%) were successfully processed (see Supplementary Table S1) and passed quality control for downstream analysis. Taxonomic classification, TVC, and Simpson's diversity index are shown longitudinally for each drain (Supplementary Tables S1–S7).

### Total viable counts across the study period

Median TVC (cfu/mL) was calculated for each sample during the three study phases (Figure 1; Supplementary Table S2). The inter-drain variation was wide. For instance in the baseline phase the median TVC in bed 14 was lowest at 1324 cfu/mL compared to bed 9 where it was highest at 8921 cfu/mL. The intra-drain variation in TVC was wide across repeated sampling – in the baseline phase the interquartile range was lowest at 1143 cfu/mL for bed 9 and highest at 5256 cfu/mL for bed 5.

**Figure 1:**
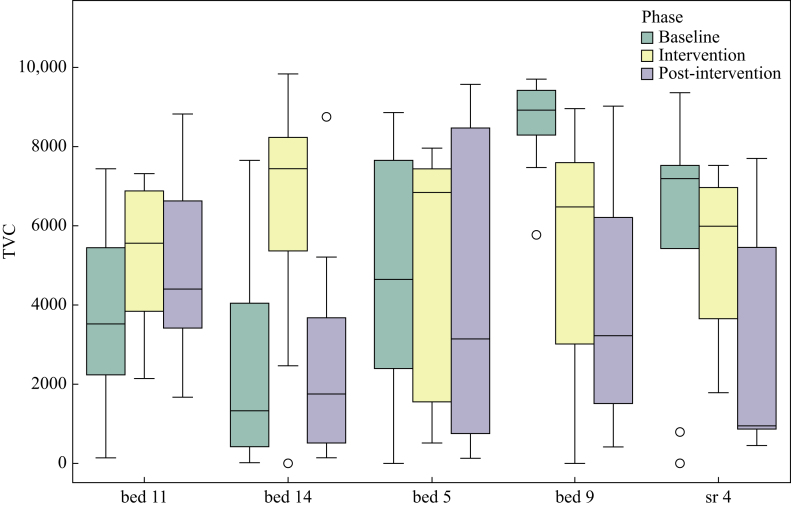
Box plots showing the distribution of total viable counts (TVC) for each drain across the three study phases.

Overall, across all drains there was no significant change in TVC between the baseline phase and intervention phase (+386 cfu/mL, SE 705, *P* = 0.59) or between the baseline phase and post-intervention phase (–1150 cfu/mL, SE 738, *P* = 0.12). Statistical analysis of the individual drains was also performed. Bed 9 showed no significant change between baseline phase and intervention phase (–2480 cfu/mL, SE 1441, *P* = 0.10), but did show a significant decrease between baseline and post-intervention phase (–4471 cfu/mL, SE 1441, *P* = 0.006). Similarly, sr 4 showed no significant change in TVC between baseline and intervention phase (–923 cfu/mL, SE 1404, *P* = 0.52), but did show a significant decrease in TVC between baseline and post-intervention phase (–3186 cfu/mL, SE 1462, *P* = 0.044). There were no significant changes seen in the TVC of bed 5, bed 11, and bed 14 across the study periods.

### Drainome diversity across the study periods

Intra-drain drainome diversity (Supplementary Table S1) was compared across the three study phases for all samples, showing a small but significant increase in diversity between the baseline phase and intervention phase (–0.07 in Simpson's index, SE 0.03, *P* = 0.007). However, there was no change in drainome diversity between baseline and post-intervention phase (–0.05 in Simpson's index, SE 0.03, *P* = 0.08), suggesting that any effect of decontamination was not sustained.

### Changes in proportion of bacterial genera in the intervention and post-intervention phases

Changes in the proportions of certain genera across the study periods were assessed (Figure 2, Supplementary Figures S1–S4). Six bacteria that were commonly found amongst the top genera in every sample, namely *Pseudmonas* sp., *Acidovorax* sp., *Diaphorabacter* sp., *Acinetobacter* sp., *Rhizobium* sp., and *Citrobacter* sp., were identified (Supplementary Table S8). *Aeromonas* sp. was found to be one of the most common organisms in four drains, and *Klebsiella* sp. in three drains. The change in proportion of these genera between study periods is shown in Table I.

**Figure 2:**
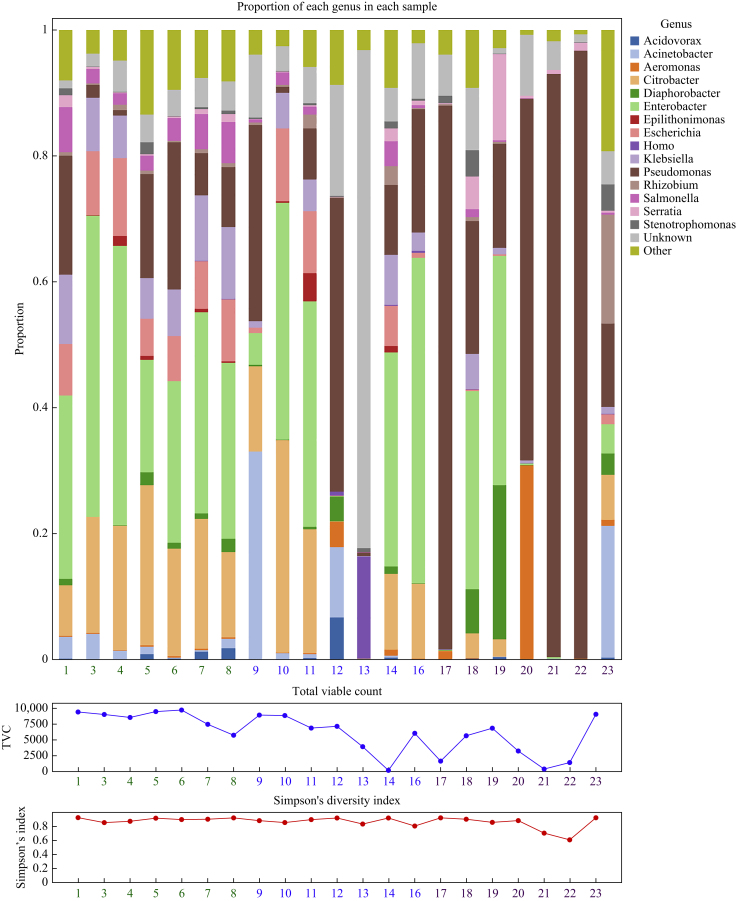
Stacked bar chart showing relative abundance of microbial genera in each sample from bed 9 (upper panel), longitudinal total viable count (TVC) data (middle panel, cfu/mL) and microbial diversity as measured by Simpson's diversity. Sample numbers (*N* = 21) on the *x*-axis are coloured according to study phase: baseline, green; intervention, blue; post-intervention, purple.

**Table I:** Change in bacterial genera across study phases

Genus	Baseline		Intervention			Post-intervention		
	(%)	SE (%)	(%)	SE (%)	*P*-value	(%)	SE (%)	*P*-value
*Pseudomonas*	18.3	6.2	40.5	5.7	<0.001	47.6	6.0	<0.001
*Acidovorax*	5.2	1.1	1.2	0.9	<0.001	2.0	1.0	0.001
*Diaphorobacter*	4.4	0.9	1.3	0.8	<0.001	2.0	0.8	0.004
*Acinetobacter*	4.5	1.5	4.9	2.1	0.85	2.7	2.2	0.41
*Rhizobium*	1.4	0.4	0.6	0.5	0.16	1.4	0.6	0.96
*Citrobacter*	5.3	2.5	11.3	2.6	0.02	6.3	2.7	0.7
*Aeromonas*	3.2	1.3	1.7	1.8	0.42	3.3	1.9	0.95
*Klebsiella*	3.4	1.2	2.9	1.7	0.75	3.8	1.9	0.84

The proportions of *Pseudomonas* sp. were significantly higher in the intervention phase compared to the baseline phase (18.3% to 40.5% (+22.2%), SE 5.7%, *P* < 0.001) and in the post-intervention phase (18.3% to 47.6% (+29.3%), SE 6.0%, *P* < 0.001) compared with baseline. The change in *Pseudomonas* sp. proportions was not driven by a change in *P. aeruginosa*, which saw no significant change across intervention periods. Between the baseline and intervention phase the proportion of *P. aeruginosa* did not change (7.3% to 9.9% (+2.6%), SE 2.2%, *P* = 0.2).

### Detection of antimicrobial residues

Each sample was tested for presence of antimicrobial residues of β-lactams, tetracyclines, sulfonamides, and quinolones (Supplementary Table S1). Of the 115 samples, antimicrobial residues were found in 31 (27%) samples, in all sinks: bed 5 (Supplementary Figure S5, 5/115, 22%), bed 9 (Figure 3, 8/23, 35%), bed 11 (Supplementary Figure S6, 6/23, 26%), bed 14 (Supplementary Figure S7, 8/23, 35%), and sr 4 (Supplementary Figure S8, 4/23, 17%), and across all intervention periods (baseline 10/40 (25%), intervention 16/40 (40%), post-intervention 5/35 (14%)). Residues of β-lactams were found in 19/115 (17%) samples, quinolones in 15 (13%) samples, sulfonamides in three (3%) samples, and tetracyclines in two (2%) samples. There was no significant difference between the presence of antimicrobial residues between the baseline and intervention (*P* = 0.08), nor between the baseline and post- intervention phase (*P* = 0.6).

**Figure 3:**
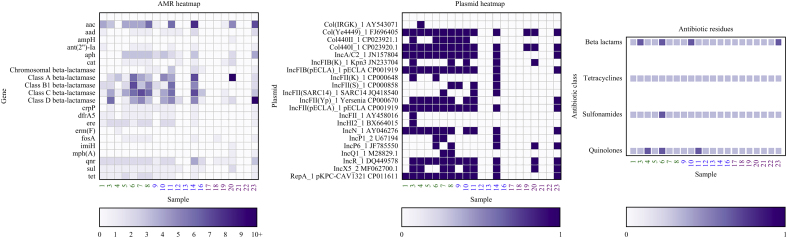
Longitudinal sampling data for bed 9, showing presence or absence of antimicrobial resistance genes (left), detection of plasmids (middle), and detection of antibiotic residues (right). Antimicrobial resistance genes are presented in gene families where more than one gene of that family is present. Sample numbers on the *x*-axis are coloured according to study phase: baseline, green; intervention, blue; post-intervention, purple.

### Analysis of antimicrobial resistance genes and plasmids

Data for the detection of ARGs and plasmids are shown in Figure 3, Supplementary Table S9, and Supplementary Figures S5–S8. ARGs were highly prevalent in the samples across the study period; ARGs encoding extended-spectrum β-lacatamases were found in all samples during the study period. NDM-carbapenemases were found in six samples from three drains.

## Discussion

Here we present a workflow for characterization of the drainome from hospital drainwater samples. By applying it longitudinally, we could evaluate the effect of decontamination procedures, both in terms of changes in estimated TVCs as well as change in bacterial community structure, diversity and prevalence of ARGs. Other methods to assess decontamination interventions are limited, many of which require laborious culture and focus on a limited range of potential pathogens rather than the total population of organisms [11].

In our study, no difference in total viable count was seen over the study periods, suggesting that the decontamination intervention had limited effect. The failure to show large and significant effect sizes for this measure may suggest that decontamination protocols could be optimized.

Despite the small change in overall diversity, there were some major shifts in community structure, which included a significant increase in *Pseudomonas* sp.; this was accompanied by significant decreases in *Acidovorax* sp. and *Diaphorobacter* sp. This suggests that peracetic acid had influenced the drainome composition; however, since our study did not include any sinks without intervention over the same time period, it is difficult to attribute the changes to the effect of peracetic acid with confidence, rather than natural evolution without intervention. Species-level data are available from this workflow, as demonstrated with analysis of *Pseudomonas aeruginosa*, to allow more granular assessment of drainome changes with reference to specific pathogenic organisms.

*Pseudomonas* sp. has been noted to be relatively resistant to decontamination by peracetic acid, potentially related to the requirement for higher concentrations to allow penetration and degradation of biofilm [20,21]. There may be other groups of bacteria that are more or less susceptible to the effects of peracetic acid, resulting in selection of particular strains. Other disinfecting agents may show improved effectiveness in decontamination of *Pseudomonas* sp. [22].

These findings are in contrast to our prior study in a different clinical environment in the same hospital that showed a modest but statistically significant and sustained decrease in TVC of approximately one-third, as measured by the same non-culture-based technique [23]. The reason for this disparity is unclear, but there are differences in the age and design of the handwash basins and drains in ICU compared to the wards where the previous study was conducted. The sink design, as shown in a photograph in the Supplementary Methods, is commonly used in healthcare settings and complies with the specification detailed within Health Building Note 00–10 [24]. The design of the handwash basin and drainage system is likely an important factor in determining the effectiveness of disinfectant products, e.g. if the disinfectant product is not able to remain in contact with biofilms for sustained periods required to fully decontaminate the area [25]. Prior studies have associated poor sink design and placement within clinical areas with nosocomial outbreaks and have suggested that a stop valve to increase disinfectant exposure can improve decontamination [26,27]. Other products are available in a foam formulation, which may aid distribution and retention of the product. This underlines that data are also required from other wards to ascertain whether the effects seen here are sink-specific. Sinks without enhanced decontamination could also be sampled to assess drainome changes in the absence of intervention.

Ultimately handwash basins, showers, drains, and associated fixtures will continue to be a risk to vulnerable patients, with removal of outlets to provide water-free care being the most effective strategy to mitigate risk [10].

Detection of antimicrobial residues in sink drains was common and likely corresponds to high rates of patient antimicrobial consumption in a high-acuity setting such as the ICU. This finding suggests that clinical handwash basins are being used other than for their intended purpose (washing hands), for example disposal of bodily fluids and/or unused antimicrobials. Previous work has shown that clinical handwash basins in a medical ICU were used for handwashing for only 4% of total interactions over a 60-day period [28]. Such underuse for handwashing may reflect reliance on alcohol-based hand rub as per WHO guidelines [29]. This may facilitate emergence of antimicrobial resistant strains and persistence of antimicrobial resistance genes in the environment.

As expected and congruent with other studies, drainwater samples were a rich reservoir of ARGs, particularly those encoding resistance to β-lactams and other clinically relevant classes. NDM-carbapenemases are rarely identified in our cohort of patients, and similarly were identified in only three drains in six samples.

The workflow captured bacterial genomes, but fungal organisms were found at low abundance, for instance, with only 912 reads classified as *Aspergillus* sp. and 174 reads classified as *Candida* sp. Low read counts for fungal pathogens have been seen in our clinical metagenomic studies, which may reflect workflow-specific effects or the low biomass of fungal pathogens in comparison with bacterial counterparts [15]. In addition, the workflow described here does not detect RNA organisms such as viral pathogens which may be found in wastewater, though upcoming workflows may offer a solution for the combined detection of DNA and RNA organisms [30,31]. In addition, there were many detections of ARGs and plasmids but with coverage beneath our previously validated threshold for reporting. These could represent partial detection of genes or elements that would otherwise not be reported. Moreover, genes or elements with low nucleotide identity to known plasmids could represent novel antimicrobial resistance mechanisms of particular interest.

Future studies should focus on assessing the effectiveness of other decontamination strategies, perhaps during ongoing outbreaks of resistant organisms where hospital sinks can act as reservoirs of the organism [[6], [7], [8]]. Our ability to detect ARGs conferring resistance to carbapenems suggest that metagenomic study of the drainome may have utility for this purpose. In addition, further prospective monitoring of the drainome may allow us to identify environment-to-patient transmission if similar organisms and ARGs newly appear in recently admitted patients. Other measures of the effectiveness of decontamination would also be valuable to measure, such as change in drug-resistant pathogens and incidence of nosocomial infection.

It is important to evaluate and compare the effectiveness of decontamination interventions to justify expenditure of health economic resource. The optimal strategy for reduction of transmission from sinks is not known; however, this methodology can be used to assess the effectiveness of decontamination in a culture-independent manner [32]. Measurements of the drainome can provide microbiological assessment, but further studies judging decontamination effects on microbiological and clinical outcomes are required.

## Conflict of interest statement

Professor J. Edgeworth is employed part-time by Oxford Nanopore Technologies as VP Medical Affairs.

## Funding

L.B.S. and G.N. receive funding from the Medical Research Council (L.B.S.: MR/W025140/1; G.N.: MR/T005416/1).

## Ethical statement

The study was classed as research not requiring NHS Research Ethics Committee (REC) review and did not include human participants. It was registered and approved under locally defined procedures as a laboratory-supported environmental study.

## Data availability

Depersonalized metagenomic data are available from the Sequencing Read Archive (BioProject PRJNA1090773).

## Figures and Tables

**figs1:**
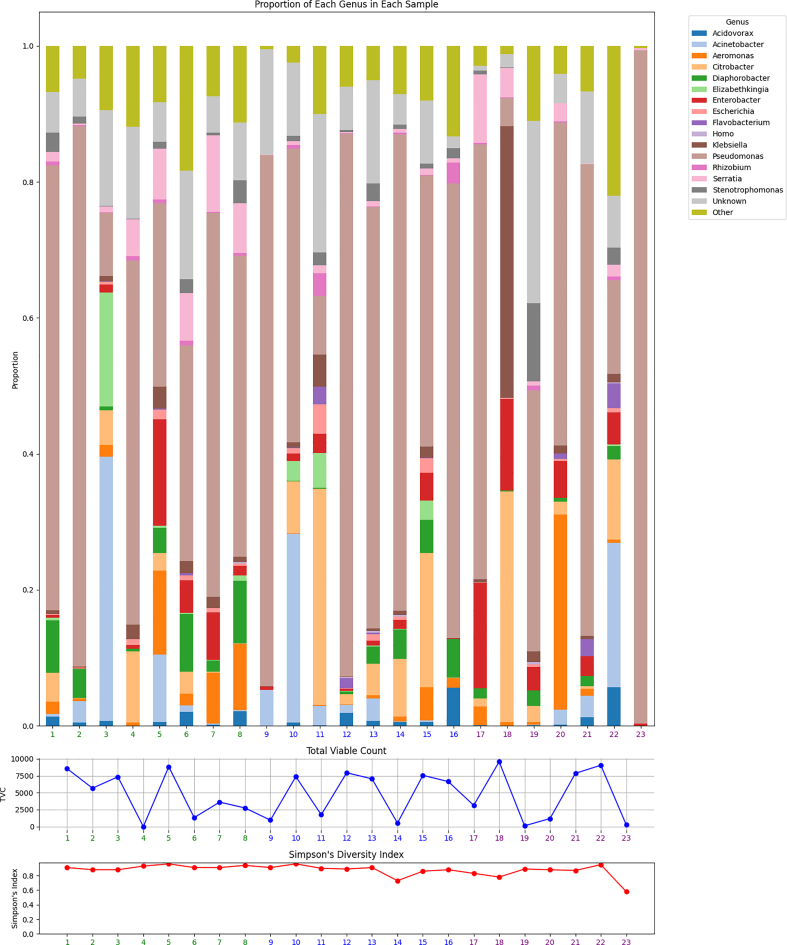


**figs2:**
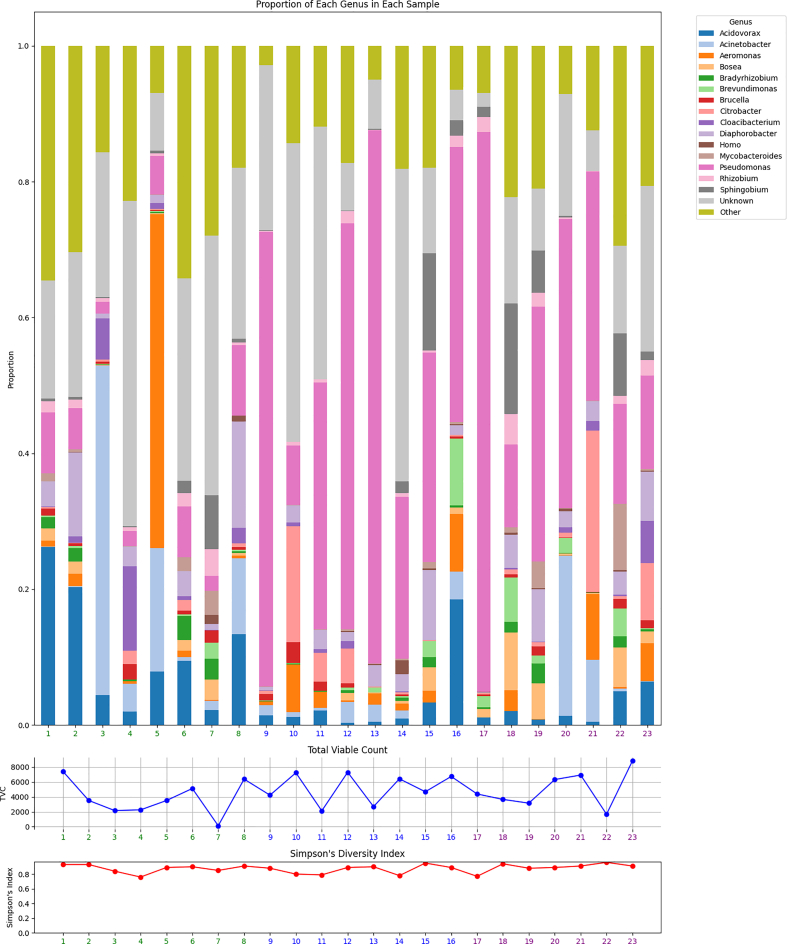


**figs3:**
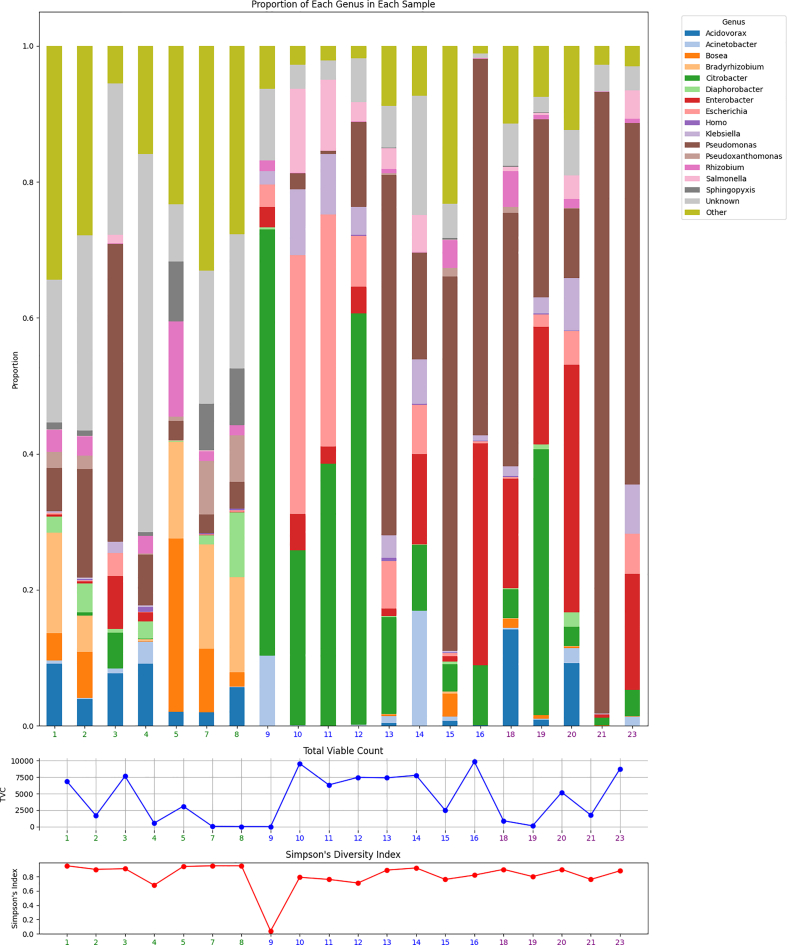


**figs4:**
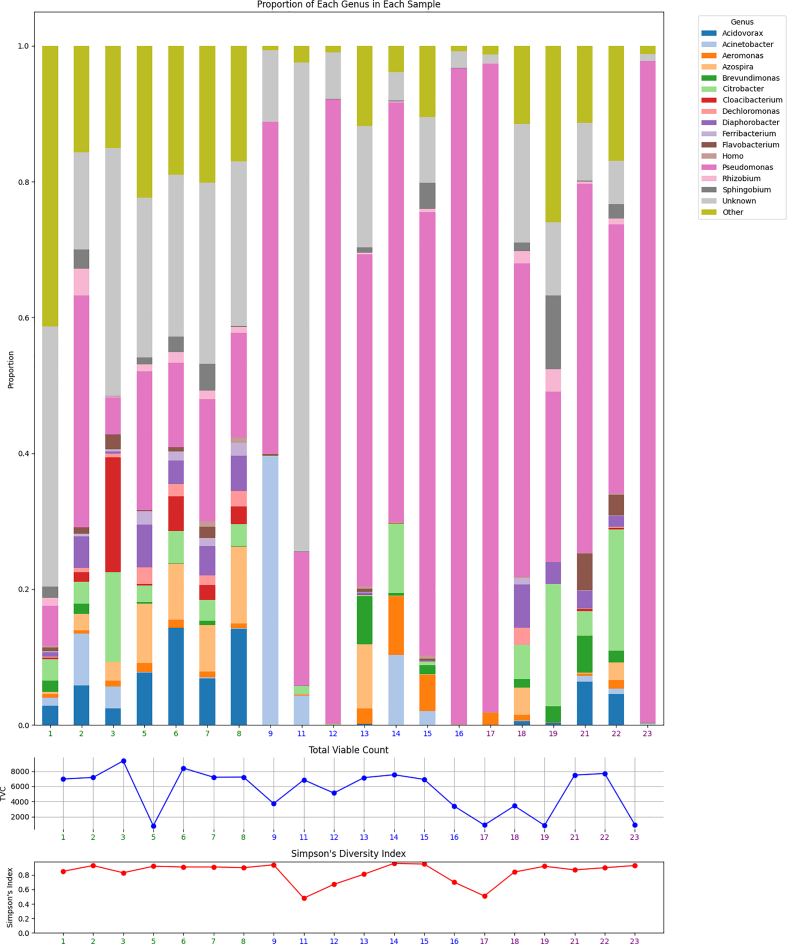


**figs5:**
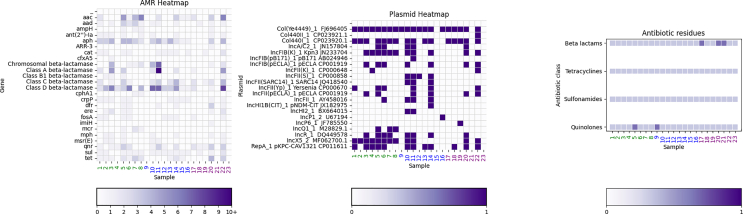


**figs6:**
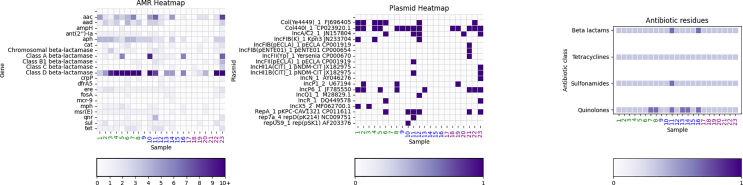


**figs7:**
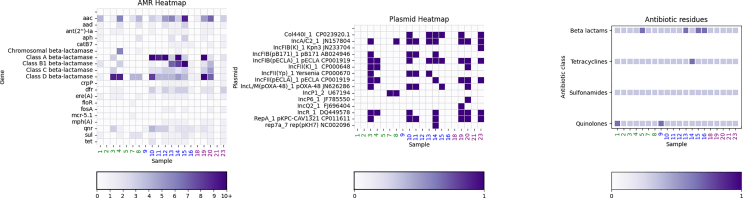


**figs8:**