# Supplementary material for: The drainome: longitudinal metagenomic characterization of wastewater from hospital ward sinks to characterize the microbiome and resistome and to assess the effects of decontamination interventions
Source: J Hosp Infect. 2024 Nov;153:55–62. doi: 10.1016/j.jhin.2024.06.005 (PMC11825382; doi:10.1016/j.jhin.2024.06.005)

**The drainome: longitudinal metagenomic characterisation of wastewater from hospital ward sinks to characterize the microbiome and resistome and assess effectiveness of decontamination interventions.**

**Supplementary Methods**

Representative photograph of the five hospital ward sinks sampled during the study.


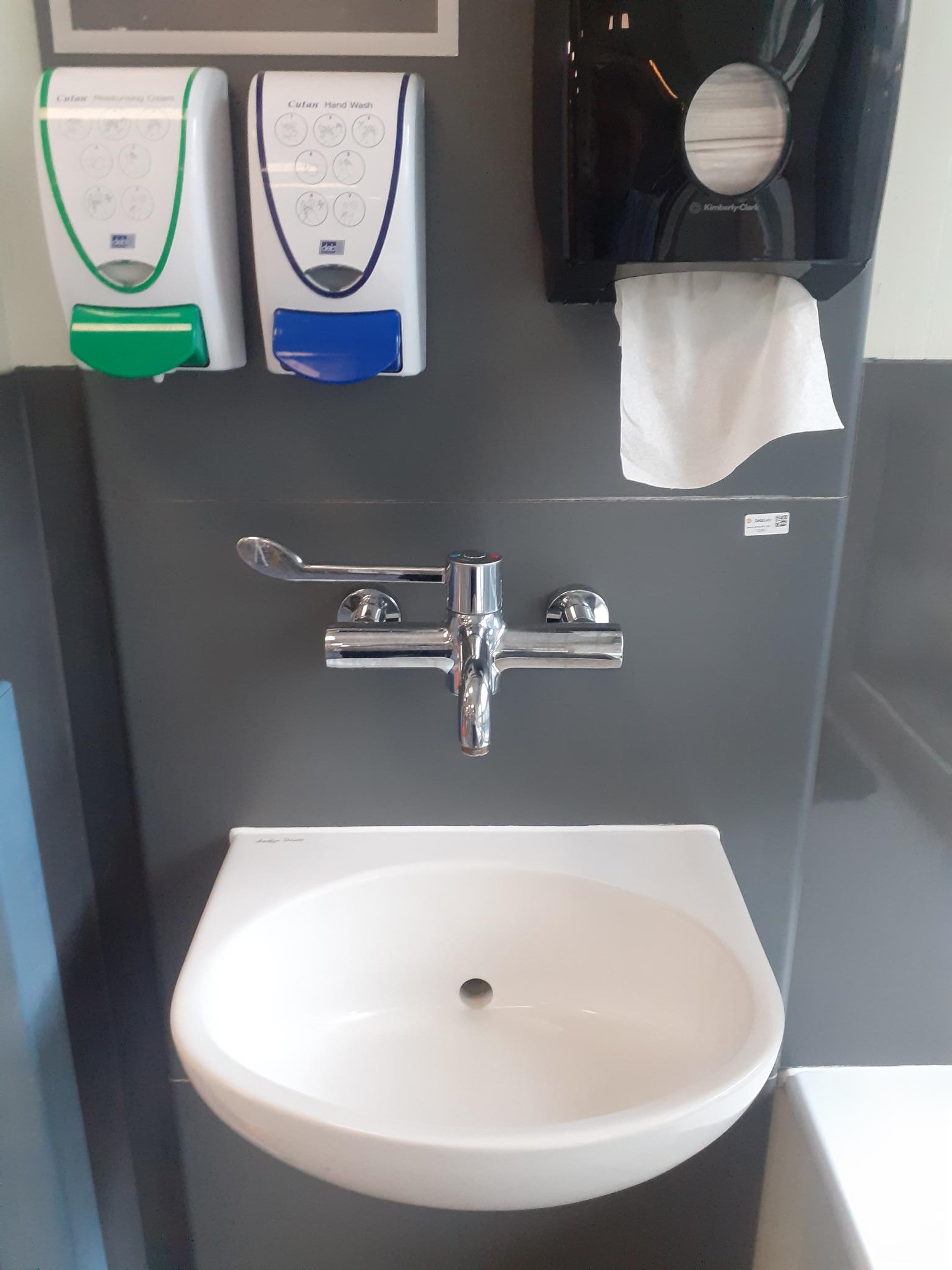

Supplement: Multimedia component 1 [file mmc1.docx]
